# Analysis of Retinal Architectural Changes Using Intraoperative OCT Following Surgical Manipulations With Membrane Flex Loop in the DISCOVER Study

**DOI:** 10.1167/iovs.17-21584

**Published:** 2017-07

**Authors:** Atsuro Uchida, Sunil K. Srivastava, Justis P. Ehlers

**Affiliations:** 1 Ophthalmic Imaging Center, Cole Eye Institute, Cleveland Clinic, Cleveland, Ohio, United States; 2 Cole Eye Institute, Cleveland Clinic, Cleveland, Ohio, United States

**Keywords:** intraoperative OCT, epiretinal membrane, macular hole, surgical instrument

## Abstract

**Purpose:**

We investigate acute retinal alterations identified on intraoperative optical coherence tomography (iOCT) immediately following surgical intervention with the Finesse Flex Loop for vitreoretinal interface disorders.

**Methods:**

The Determination of feasibility of Intraoperative Spectral domain microscope Combined/integrated OCT Visualization during En face Retinal and ophthalmic surgery (DISCOVER) study is a prospective multisurgeon intraoperative OCT clinical study. Subjects who had participated in the DISCOVER study and had undergone macular surgery with the membrane flex loop from August 2014 to July 2016 were identified. iOCT images and video sequences were evaluated at various surgical time points. Subjects were excluded if iOCT images were not obtained over the area of membrane peeling performed with the membrane flex loop. Qualitative characteristics of intraoperative changes in retinal images were analyzed, with particular focus on the retinal layers within the bed of membrane peeling performed specifically with the membrane flex loop.

**Results:**

We studied 34 eyes of 34 patients, with a mean age of 72.7 (± 6.4) years, 25 of whom were women. The intraoperative diagnosis was full thickness macular hole in 21 eyes (62%) and epiretinal membrane in 13 (38%). All eyes had successful indocyanine green–assisted inner limiting membrane (ILM) flap initiation using the membrane loop. Intraoperative OCT demonstrated expansion of the ellipsoid zone-to-RPE distance in 3 eyes (9%), definitive subretinal fluid accumulation in 1 eye (3%), and hyperreflectivity of the inner retinal layers associated with retinal hemorrhage in 10 eyes (29%). Retinal breaks were not observed in any eye.

**Conclusions:**

Acute retinal alterations after ILM peeling with the membrane flex loop were visualized at a frequency of less than 10%. Additional research is needed to understand the clinical impact, if any, of these architectural alterations.

Over the past several years, intraoperative optical coherence tomography (iOCT) has been providing new insight into the changes to retinal anatomy during macular surgery.^1–7^ Our group and others have demonstrated that following surgical intervention (e.g., membrane peeling, release of the hyaloid in vitreomacular traction syndrome) alterations may be identified frequently in the retinal anatomy.^1–7^ These alterations often are located in the outer retina and include changes in the distance between the ellipsoid zone (EZ; i.e., inner segment/outer segment junction [IS/OS]) and the RPE as well as the cone outer segment tips-to-RPE distance, resulting in microarchitectural changes demonstrated by subtle increased subretinal hyporeflectivity.^1–5,8^ Additionally, full-thickness retinal elevation has been noted with accumulation of subretinal fluid. Many of these alterations have been linked clearly to instrument-tissue interactions, such as direct “pinch-and-peel” with the vitreoretinal and the diamond-dusted membrane scraper.^3^ The data remain unclear regarding the potential instrument-dependent role in these alterations and there is some suggestion that the type of instrument used may have a differential impact on the retinal tissue.^3^ The membrane loop (FINESSE Flex loop; Alcon, Fort Worth, TX, USA) is a recently Food and Drug Administration (FDA)–cleared membrane peeling instrument designed for internal limiting membrane (ILM) peeling and the impact of the membrane loop on the underlying retinal tissues remains unknown. We evaluated the retinal architecture with iOCT following membrane peeling procedures with the membrane loop.

## Methods

The Determination of feasibility of Intraoperative Spectral domain microscope Combined/integrated OCT Visualization during En face Retinal and ophthalmic surgery (DISCOVER) study is an Internal Review Board (IRB)-approved prospective study examining the feasibility and use of microscope-integrated iOCT during ophthalmic surgery.^1^ The overall methods of the procedures have been described previously.^1^ Informed consent was obtained from all participants.

Among participants enrolled in the DISCOVER study,^1^ patients who had surgical intervention for epiretinal membrane (ERM) or full-thickness macular hole (FTMH) from August 2014 to July 2016 were identified. Of those, eyes that underwent membrane peeling procedures using the membrane loop were identified and included in this analysis. Intraoperative imaging for this portion of the DISCOVER study was performed using a prototype Rescan 700 (Carl Zeiss Meditec, Oberkochen, Germany) iOCT system integrated on the Lumera 700 (Carl Zeiss Meditec) microscope platform. The Rescan 700 is a real-time intraoperative spectral-domain optical coherence tomography (SD-OCT) equipped with an 840 nm wavelength light source, 5.5 μm axial resolution in tissue, and scanning speed of 27,000 A-scans per second. An A-scan depth is 2 mm and scan length is adjustable from 3 to 16 mm. It includes Z-tracking, focus control for image stabilization, and quality control. The microscope provides real-time OCT imaging and video recording overlay during the surgery directly in the surgical microscopic view. The Resight (Carl Zeiss Meditec) wide-angle viewing system or magnified contact lens was used for surgical and iOCT visualization. Exclusion criteria included lack of iOCT images in the area of membrane flex loop peeling or insufficient iOCT quality for interpretation.

### Surgical Procedure

All subjects underwent standard 3-port pars plana vitrectomy (23- or 25-gauge). Phacoemulsification with simultaneous intraocular lens implantation for cataract formation was performed, if indicated. After completion of the core vitrectomy, the posterior hyaloid was elevated carefully using the vitreous cutter for subjects without posterior vitreous detachment. In all cases, indocyanine green (ICG) was used to stain the membranes. For ERM and FTMH cases, the peel was initiated by elevating an edge of presumed ILM. Generally, an initial flap of ILM was created with a membrane loop starting inferior to the fovea close to the vascular arcade. For ERM cases, the location of ILM flap initiation was selected carefully using a combination of the iOCT findings and identifying the area of maximal ICG staining. Once an ILM flap has been created, iOCT could be used to visualize the characteristic “curl” that is noted with elevated ILM intraoperatively. The remaining membrane peel was completed based on surgeon preference with either vitreoretinal forceps, suction with a vitrectomy probe, or the membrane loop. Before switching peel techniques, an iOCT image was obtained in the bed of the ILM that had been elevated with the membrane loop.

### Intraoperative OCT Snapshots/Video Acquisition

Based on the DISCOVER study protocol, iOCT imaging was obtained at various surgeon-selected surgical milestones, including immediately before surgical manipulation, immediately before membrane peeling (subsequent to hyaloid separation), during the course of macular manipulation (subsequent to ILM flap lifting and between membrane peeling procedures), and following membrane peeling procedures. The iOCT snapshots/video were recorded in a horizontal and/or vertical orientation.

### Intraoperative and Clinical OCT Image Analysis

Each subject's imaging dataset (e.g., surgical video, iOCT video files, iOCT macular cubes, and iOCT snapshots) was reviewed carefully by an independent masked expert OCT reader. Imaging was analyzed at three surgical points: iOCT acquired immediately before membrane peeling (prepeel scan), after initial flap elevation and propagation with the membrane loop (postflap), and after complete membrane removal (postpeel). For this report, snapshots/videos/macular OCT cubes were reviewed for the specific anatomic location where the membrane loop was used with real-time and static iOCT. The prepeel iOCTs were reviewed for any preexisting retinal abnormalities. The postflap and postpeel iOCTs then were reviewed with focusing on the retinal layers within the bed of membrane loop peeling.

The primary endpoint was to evaluate immediate intraoperative alterations to the retina (e.g., increased subretinal hyporeflectance, new subretinal fluid) within the bed of peeling where the membrane loop was used during surgical interventions. OCT interpretation included qualitative review of the OCT features with particular focus on the retinal layers, including disruption/attenuation, expansion of the EZ-to-RPE distance (e.g., increased subretinal hyporeflectance), and full-thickness retinal elevation with subretinal fluid accumulation, retinal break development, increased inner retinal hyperreflectivity associated with retinal hemorrhage, and increased inner retinal hyperreflectivity not associated with retinal hemorrhage.

In addition to the iOCT imaging data, presurgical SD-OCT images were reviewed for preoperative abnormalities in all eyes that did not have prelift/prepeel iOCT imaging. In those eyes that required hyaloid elevation and demonstrated postpeel abnormalities, the presurgical SD-OCT was reviewed for preexisting abnormalities and to further characterize the hyaloid-retina relationship. Postoperative SD-OCT scans were reviewed for all eyes demonstrating postpeel abnormalities for any persistent retinal abnormalities.

## Results

### Clinical Characteristics and Demographics

A total of 44 eyes of 44 patients identified initially from the DISCOVER study underwent membrane/ILM peeling with membrane loop for vitreomacular interface disorders. Ten eyes were excluded from the study either due to poor quality of the iOCT image to evaluate any of the parameters (3/44 eyes, 7%) or lack of postpeel iOCT scans clearly within the bed of the membrane flex loop peel zone (7/44 eyes, 16%). Of the remaining 34 eyes of 34 patients, the mean age was 72.7 ± 6.4 years (mean ± SD; range, 59–84 years). There were 25 women (74%) and 9 men (26%). A total of 21 eyes (62%) were treated for FTMH and 13 (38%) for ERM. The mean preoperative visual acuity (VA) was 20/70, (range, 20/25–20/200). Of 23 (68%) phakic eyes, 20 (87%) underwent combined cataract and vitrectomy surgery. Clinical demographics of the patients and retinal alterations assessed with iOCT are shown in the Table.

**Table:**
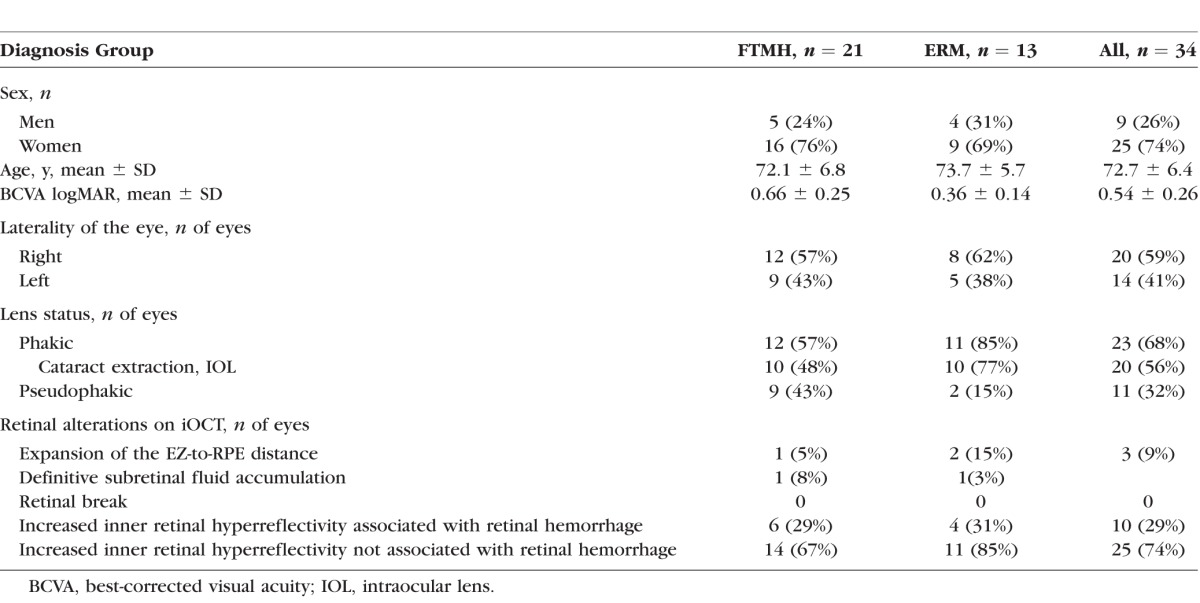
Clinical Demographics and Retinal Alterations Identified With Intraoperative OCT

### Surgical Procedure

A total of 23 eyes (68%) underwent 23-gauge vitrectomy and 11 (32%) underwent 25-gauge surgery. Preexisting complete posterior vitreous detachment was confirmed in 15 eyes (44%). Triamcinolone acetonide was used in 19 eyes (56%) to visualize the hyaloid and posterior vitreous detachment was induced in 19 eyes (56%). ICG was used to stain the ILM in all eyes. For macular surgery, a wide-angle viewing system with noncontact 60 diopter (D) lens was used in 32 eyes (94%) and a magnified contact lens was used in 2 eyes (6%). The membrane loop was used to initiate the ILM flap in all cases. Once the ILM initially had been elevated and propagated, the instrument choice for removing the residual ILM was vitreoretinal forceps in 28 eyes (82%), the membrane loop in 4 (12%), and the vitrectomy probe in 2 (6%).

### Intraoperative OCT Findings

**Figure 1:**
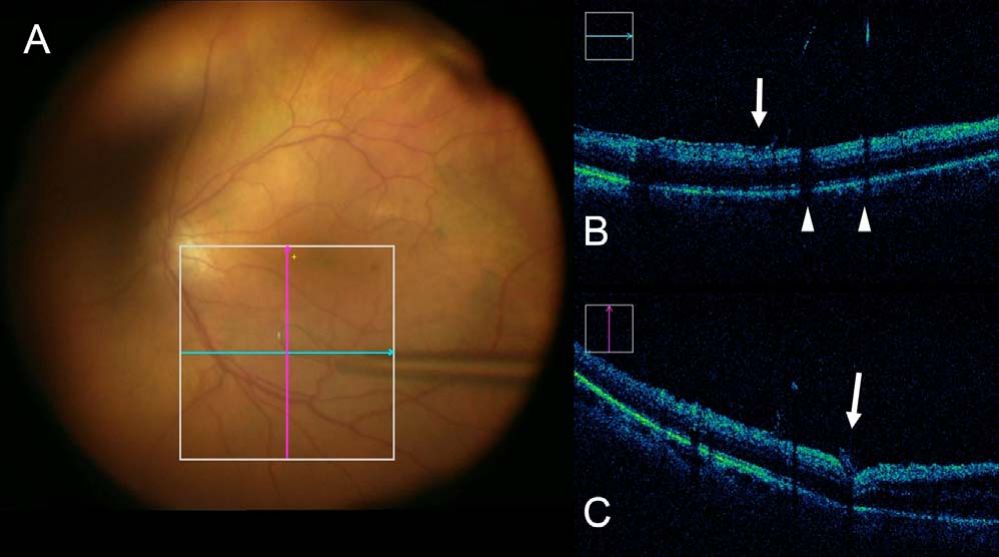
iOCT of a real-time manipulation with the membrane loop. (A) Surgical video view during ILM peel with the membrane loop. Blue and pink lines show approximate section of images in (B) and (C), respectively. (B) Horizontal B-scan of the posterior pole shows shadowing artifact posterior to the metallic membrane loop (white arrowhead). Lifted edge of the residual ILM before surgical removal can be seen (white arrow). (C) In vertical B-scan, the membrane loop on the retina surface compressing the neurosensory retina is seen at the area of the tip (white arrow). Minimal shadowing from the metallic loop is visible (Suppplementary Video).

Surgical manipulation with the membrane loop was imaged successfully with iOCT. During ILM peel, the membrane loop sweeping across the retinal surface was observed clearly, as well as partial compression of the retinal tissues at the area where the tip was placed on iOCT (Fig. 1, Supplementary Video). The shadowing created by this metallic instrument minimally limited views of the iOCT on B-scan. In relation to microarchitectural change, expansion of the EZ-to-RPE distance (e.g., increased subretinal hyporeflectivity) was observed in 3 eyes (9%; 1 FTMH eye, 2 ERM eyes). In all 3 cases, the loop was used to initiate the peel and to develop the flap in 30% to 70% of the entire peel area. The forceps then were used in each case to complete the peel. The postpeel OCT was used before completing the peel with the forceps.

**Figure 2:**
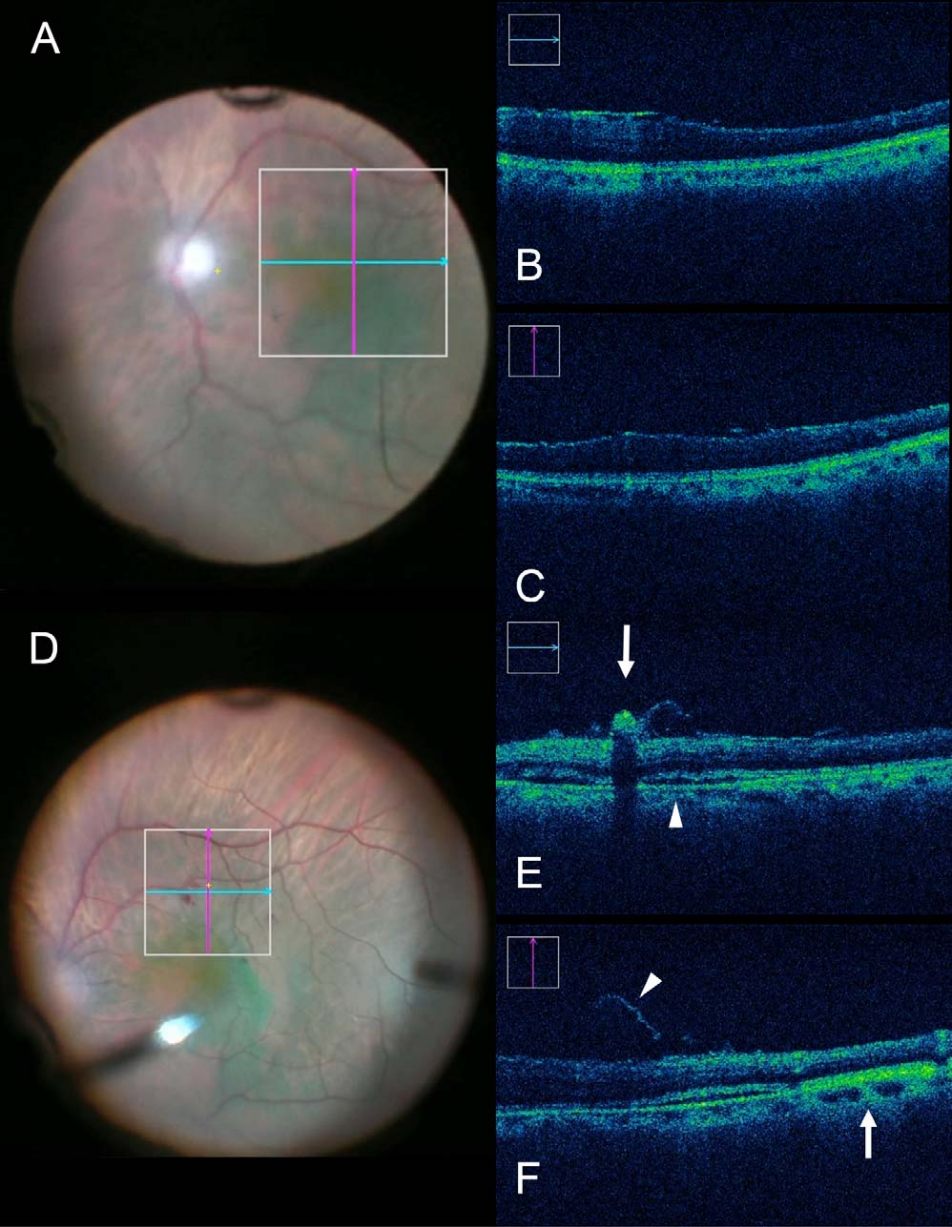
iOCT immediately before and following membrane loop manipulation during ERM removal. (A) Intraoperative surgical view before membrane peeling. ILM is stained with ICG. (B, C) Neither definitive subretinal fluid accumulation nor expansion of the EZ to RPE distance is visible. (D) Intraoperative surgical video view following elevation of ILM and ERM with the membrane loop. (E) Increased inner retinal hyperreflectivity associated with retinal hemorrhage can be seen (white arrow). Definitive subretinal fluid accumulation also is visible (white arrowhead). (F) Expansion of the EZ to RPE distance can be seen (white arrow) as a large area of subretinal hyperreflectance adjacent to the subretinal fluid. Lifted ILM is evident (white arrowhead).

**Figure 3:**
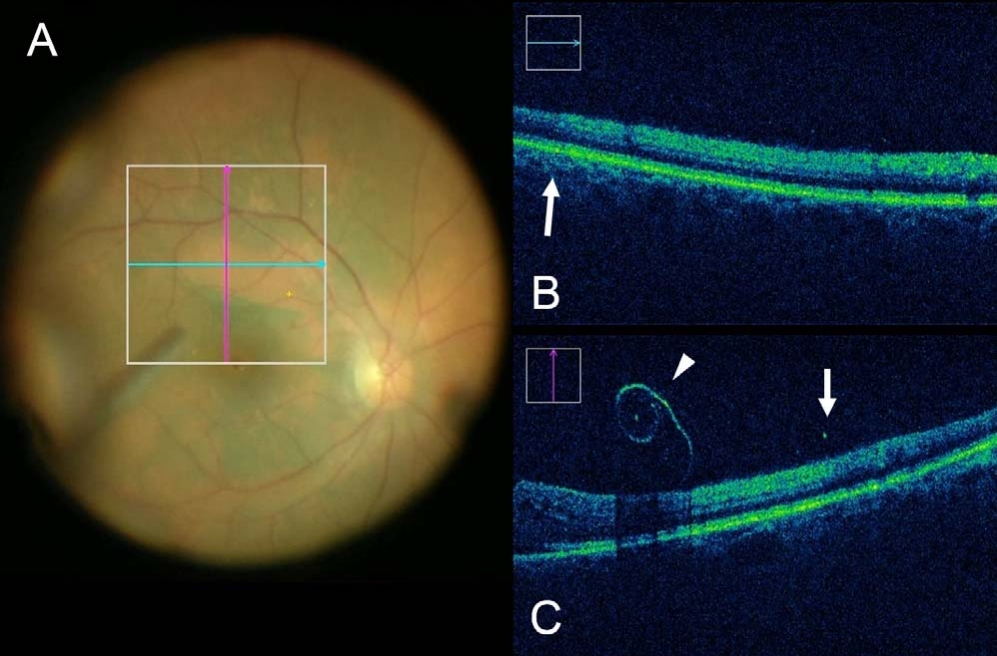
iOCT following elevation of the ILM with the membrane loop. (A) Intraoperative surgical video after creating the ILM flap. (B) Transition in signal strength is noted at area with ICG–stained ILM (arrow). (C) Hyperreflectance of ICG-stained ILM can be seen (white arrowheads) with increased underlying shadowing of tissues. Junction between residual ILM and peeled area is differentiated clearly based on signal strength within the retina (white arrow).

Full-thickness retinal elevation with subretinal fluid accumulation was observed in 1 eye with ERM (Fig. 2). In this case, video review showed that the membrane loop was used several times to elevate a particularly adherent ILM located in the inferior macula. No retinal breaks within the area membrane/ILM peeled were observed in any eye. Through reviewing surgical videos, postpeel mild focal retinal hemorrhages were confirmed in 13 eyes (38%) within the area the membrane loop was used. Increased inner retinal hyperreflectivity associated with retinal hemorrhage was confirmed in all eyes that had corresponding iOCT scans in the area of hemorrhage following peeling (10 eyes). The hemorrhages originated from using membrane flex loop were mostly small and localized. Following removal of ICG-stained ILM, increased signal strength of the retinal tissue was noted in 25 eyes (74%; Fig. 3). These findings were observed immediately after ILM had been peeled.

### Preoperative and Postoperative Clinical SD-OCT Review

Among eyes that demonstrated alterations on postpeel iOCT scans, 9 eyes underwent hyaloid elevation before peeling. Presurgical SD-OCT was reviewed for all of these cases. All cases demonstrated either perifoveal posterior vitreous detachment (PVD) or complete vitreous separation at the macula region before surgical intervention, and no findings were associated with the location of the postpeel iOCT abnormalities.

For all eyes demonstrating postpeel abnormalities, the first postoperative SD-OCT scan was reviewed for any persistent abnormalities. For all cases, the intraoperative abnormalities noted on iOCT had resolved at the time of the first postoperative SD-OCT assessment. All available scans were reviewed for abnormalities, including raster and macular cube scans.

## Discussion

In this study, we reported the intraoperative retinal alterations identified with iOCT for vitreomacular interface disorders managed with the membrane loop. The movement of this instrument and underlying retinal interface interactions were visualized clearly on iOCT. In this case series, 9% of eyes demonstrated microarchitectural changes identified as increased subretinal hyporeflectivity. Macroarchitectural change on OCT, identified as clear subretinal fluid, was noted in 1 eye. None of the 34 eyes showed existence of retinal breaks.

Peeling the ILM has become a frequently performed procedure during MH repair and ERM surgery; however, because the ILM is thin and transparent, atraumatic initiation of an ILM flap can be technically challenging, even with an aid of vital dyes.^9^ Various surgical techniques have been described, including “pinch-and-peel” techniques and membrane engagement with a diamond-dusted membrane scraper. Retinal architectural alterations and tissue trauma have been reported as intraoperative complications related to ILM peeling.^10,11^ Recently, a new instrument was introduced to assist surgeons with membrane peeling. The membrane loop uses a nitinol metallic thin loop with small tines for membrane engagement. The retractable loop allows for modulation of rigidity. The overall impact of this instrument on tissue alterations during surgeries remains unknown.

One previous study demonstrated that membrane peeling with either forceps and/or a diamond-dusted membrane scraper induced retinal alterations on iOCT in vitreomacular interface disorders.^3^ Inner retinal elevations were identified in 45 of 163 eyes (28%) as well as full thickness retinal elevations in 8 eyes (5%) associated with instrument–tissue interaction, immediately after the ILM peeling.^3^ In the current report, definitive subretinal fluid accumulation was observed in 3% of eyes and expansion of the EZ to RPE distance was noted in 9%. The clinical significance of these intraoperative anatomical alterations are not fully understood; however, expansion of the EZ to RPE distance may perhaps represent subclinical neurosensory retinal detachment or a stretching of the photoreceptors in the outer retina.^7,8^ One previous study examined the impact of the EZ-to-RPE expansion on macular hole closure, and determined that increased expansion of the EZ-to-RPE relationship was associated with more rapid anatomic normalization.^8^

Inner retinal hemorrhage was observed in 13 of 34 eyes (38%) in the area where ILM had been peeled with the membrane loop, and of those, increased hyperreflectivity associated with retinal hemorrhage was confirmed in 10 eyes (29%) on iOCT. Induced retinal hemorrhages were mostly small and localized, confined to superficial or inner retina confirmed on iOCT. Subtle superficial retinal hemorrhages are reported to be encountered in 66% to 75% of patients as ILM peeled with either a diamond-dusted membrane scraper or forceps.^12^ These hemorrhages usually are self-limited, which do not appear to have long-term implications.^13^ There have been rare reports of more serious issues during ILM peeling, such as significant intraoperative bleeding from small retinal vessel damage that may result in postoperative hyphema with high intraocular pressure.^14^ Also, subretinal bleeding is known to occur from the vein without a direct forceps injury.^15^ Potential etiologies for inner retinal hemorrhage following the ILM elevation include direct trauma to the inner retina from the membrane loop or applied traction from the ILM as it is removed from the retinal surface. The angle of approach for peeling with the membrane loop may alter the tractional forces compared to direct forceps engagement.

Relative hyperreflectivity also was observed in the retinal tissue following ILM peeling. Hyperreflectivity of the inner retina observed in our cases is related potentially to peeling of the ICG stained ILM. The infrared light absorbing properties of ICG are well known, with absorption peak wavelength at 805 to 810 nm in human tissues.^16^ Since the iOCT system used in this study operates with a light source of approximately 840 nm, relative increased shadowing of the underlying retinal tissue was noted following ICG staining. These findings potentially could be used as a guide for the extent of the fundus where ILM had been peeled intraoperatively. If relative hyporeflectivity of the underlying retina is observed on iOCT, additional peeling of the ILM may be considered.

Several limitations for this study should be acknowledged. Our study is limited by small sample size, lack of randomization, and its lack of a control group. A prospective, randomized comparative study with quantitative analysis and longitudinal postoperative assessment would be needed to better elucidate the differential effects of various instruments and the clinical impact of these retinal alterations.

In summary, the membrane loop facilitated initiation of the ILM peel and delivered clinically acceptable safety without any serious intraoperative complications during macular surgery. The iOCT demonstrated a low rate of retinal alterations following membrane peeling procedures. Further studies are warranted to evaluate the functional and histologic impact on retinal tissues with this instrument.

## Supplementary Material

Supplement 1

Supplement 1

## References

[1] Ehlers JP, Goshe J, Dupps WJ,. Determination of feasibility and utility of microscope-integrated optical coherence tomography during ophthalmic surgery: the DISCOVER Study RESCAN Results. *JAMA Ophthalmol*. 2015; 133: 1124– 1132. 26226623 10.1001/jamaophthalmol.2015.2376 PMC4936530

[2] Ehlers JP, Dupps WJ, Kaiser PK,. The Prospective Intraoperative and Perioperative Ophthalmic ImagiNg with Optical CoherEncE TomogRaphy (PIONEER) Study: 2-year results. *Am J Ophthalmol*. 2014; 158: 999– 1007. 25077834 10.1016/j.ajo.2014.07.034 PMC4250395

[3] Ehlers JP, Han J, Petkovsek D, Kaiser PK, Singh RP, Srivastava SK. Membrane peeling-induced retinal alterations on intraoperative OCT in vitreomacular interface disorders from the PIONEER study. *Invest Ophthalmol Vis Sci*. 2015; 56: 7324– 7330. 26559478 10.1167/iovs.15-17526 PMC4642608

[4] Ehlers JP, Tam T, Kaiser PK, Martin DF, Smith GM, Srivastava SK. Utility of intraoperative optical coherence tomography during vitrectomy surgery for vitreomacular traction syndrome. *Retina*. 2014; 34: 1341– 1346. 24667571 10.1097/IAE.0000000000000123 PMC4119825

[5] Ehlers JP, Xu D, Kaiser PK, Singh RP, Srivastava SK. Intrasurgical dynamics of macular hole surgery: an assessment of surgery-induced ultrastructural alterations with intraoperative optical coherence tomography. *Retina*. 2014; 34: 213– 221. 23860560 10.1097/IAE.0b013e318297daf3

[6] Nam DH, Desouza PJ, Hahn P,. Intraoperative spectral domain optical coherence tomography imaging after internal limiting membrane peeling in idiopathic epiretinal membrane with connecting strands. *Retina*. 2015; 35: 1622– 1630. 25829349 10.1097/IAE.0000000000000534 PMC4657857

[7] Ray R, Baranano DE, Fortun JA,. Intraoperative microscope-mounted spectral domain optical coherence tomography for evaluation of retinal anatomy during macular surgery. *Ophthalmology*. 2011; 118: 2212– 2217. 21906815 10.1016/j.ophtha.2011.04.012

[8] Ehlers JP, Itoh Y, Xu LT, Kaiser PK, Singh RP, Srivastava SK. Factors associated with persistent subfoveal fluid and complete macular hole closure in the PIONEER study. *Invest Ophthalmol Vis Sci*. 2015; 56: 1141– 1146. 10.1167/iovs.14-15765 PMC4329967 25525173

[9] Asencio-Duran M, Manzano-Munoz B, Vallejo-Garcia JL, Garcia-Martinez J. Complications of macular peeling. *J Ophthalmol*. 2015; 2015: 467814. 26425351 10.1155/2015/467814 PMC4573620

[10] Oshima Y, Ikuno Y, Motokura M, Nakae K, Tano Y. Complete epiretinal membrane separation in highly myopic eyes with retinal detachment resulting from a macular hole. *Am J Ophthalmol*. 1998; 126: 669– 676. 9822230 10.1016/s0002-9394(98)00180-9

[11] Kamura Y, Sato Y, Isomae T, Shimada H. Effects of internal limiting membrane peeling in vitrectomy on diabetic cystoid macular edema patients. *Jpn J Ophthalmol*. 2005; 49: 297– 300. 16075329 10.1007/s10384-005-0199-7

[12] Steel DH, Dinah C, Habib M, White K. ILM peeling technique influences the degree of a dissociated optic nerve fibre layer appearance after macular hole surgery. *Graefes Arch Clin Exp Ophthalmol*. 2015; 253: 691– 698. 25028313 10.1007/s00417-014-2734-z

[13] Kwok AK, Lai TY, Man-Chan W, Woo DC. Indocyanine green assisted retinal internal limiting membrane removal in stage 3 or 4 macular hole surgery. *Br J Ophthalmol*. 2003; 87: 71– 74. 12488266 10.1136/bjo.87.1.71 PMC1771454

[14] Brooks HL Jr. Macular hole surgery with and without internal limiting membrane peeling. *Ophthalmology*. 2000; 107: 1939– 1948; discussion 1948–1949. 11013203 10.1016/s0161-6420(00)00331-6

[15] Nakata K, Ohji M, Ikuno Y, Kusaka S, Gomi F, Tano Y. Sub-retinal hemorrhage during internal limiting membrane peeling for a macular hole. *Graefes Arch Clin Exp Ophthalmol*. 2003; 241: 582– 584. 12739175 10.1007/s00417-003-0676-y

[16] Alander JT, Kaartinen I, Laakso A,. A review of indocyanine green fluorescent imaging in surgery. *Int J Biomed Imaging*. 2012; 2012: 940585. 22577366 10.1155/2012/940585 PMC3346977

