# Supplementary material for: Analysis of Retinal Architectural Changes Using Intraoperative OCT Following Surgical Manipulations With Membrane Flex Loop in the DISCOVER Study
Source: Invest Ophthalmol Vis Sci. 2017 Jul;58(9):3440–4. doi: 10.1167/iovs.17-21584 (PMC5505120; doi:10.1167/iovs.17-21584)
Supplement: Supplement 1 [file iovs-58-07-53_s02.pdf]

Supplemental Video: Surgical video showing intraoperative optical coherence tomography imaging of real-time surgical manipulation with the membrane loop.
